# Approach for the vertical wind speed profile implemented in the UTCI basics blocks UTCI applications at the urban pedestrian level

**DOI:** 10.1007/s00484-024-02835-x

**Published:** 2024-12-03

**Authors:** Hyunjung Lee, Sookuk Park, Helmut Mayer

**Affiliations:** 1 Department of Environmental Engineering, College of Ocean Science, Jeju National University, Jeju, Republic of Korea; 2 Laboratory of Landscape Architecture, Department of Horticultural Science, College of Applied Life Science, Jeju National University, Jeju, Republic of Korea; 3 Chair of Environmental Meteorology, Albert-Ludwigs-University of Freiburg, Freiburg, Germany

**Keywords:** UTCI, Urban canopy layer, Vertical wind speed profile, Roughness length, Urban air flow patterns

## Abstract

The applicability of the Universal Thermal Climate Index (UTCI) at the urban pedestrian level is analysed in relation to the approach for the vertical wind speed profile (VWSP) used in the UTCI basics. As the UTCI refers to the human-biometeorological reference height (*z*_*h−b*_) of 1.1 m above ground level (agl), all meteorological input variables for UTCI calculations should also be originated from *z*_*h−b*_. Since the development of the UTCI considered meteorological conventions for stations routinely operated by national weather services, the wind speed (*v*) as one of four meteorological input variables is initially required at 10 m agl (*v*_*10 m*_). In order to calculate *v* at *z*_*h−b*_ from *v*_*10 m*_, the logarithmic law for the VWSP is implemented in the UTCI basics, but only for neutral atmospheric stability and a roughness length (*z*_*0*_) for short-cut grassland (*z*_*0*_ = 0.01 m). This methodological approach cannot be changed in the UTCI basics so far. The UTCI developers also recommend the application of the same logarithmic law, if *v* values at lower heights have to be extrapolated to 10 m agl. However, the use of this VWSP approach disregards basic air flow findings and principles in the urban canopy layer (UCL). If the UTCI is nevertheless applied at the urban pedestrian level, inaccuracies in the UTCI values will occur. With reference to *z*_*0*_ = 0.80 m, which is more typical for the UCL, they can be up to 7 K, as shown in the example of three different climate zones.

**Supplementary Information:**

The online version contains supplementary material available at 10.1007/s00484-024-02835-x.

## Introduction

### Development of the UTCI

The Universal Thermal Climate Index (UTCI), a thermo-physiologically significant index for the assessment of human thermal sensations outdoors (Jendritzky et al. 2012), was initiated by the Commission 6 (“On the development of a Universal Thermal Climate Index (UTCI)”) of the International Society of Biometeorology (ISB) and developed in COST (European programme “Cooperation in Science and Technology”) Action 730. The intention of the UTCI developers was to create an index for the quantification of human thermal sensations that is universally applicable (McGregor 2012). The UTCI should be thermo-physiologically significant over the entire range of human heat exchange. It should also be suitable for (i) all climate zones, seasons and scales from the micro to the macro level and (ii) for a range of different applications in human-biometeorology, such as daily forecasts and warnings of extreme heat or cold, human-biometeorological mappings, urban and regional planning issues, environmental epidemiology, and regional climate impact research (Błażejczyk et al. 2013).

Various aspects of the UTCI development are summarised in several articles published in the special issue “Universal Thermal Comfort Index (UTCI)” of the International Journal of Biometeorology (volume 56, issue 3, May 2012). The need for a “universal” thermal comfort index is explained (Jendritzky et al. 2012), characteristics of the Fiala multi-node model forming the thermo-physiological basics of the UTCI are described (Fiala et al. 2012; Psikuta et al. 2012), the adaptive human clothing model for the UTCI is outlined (Havenith et al. 2012), the operational procedure for determining the UTCI is indicated (Bröde et al. 2012), UTCI inaccuracies due to inaccuracies in determining radiant flux densities from both observed or modelled meteorological data are evaluated (Weihs et al. 2012), and the performance of the UTCI is discussed in comparison to other thermal assessment indices (Błażejczyk et al. 2012; Kampmann et al. 2012).

### UTCI basics

There are numerous publications dealing with various aspects of the UTCI, such as the special issue “UTCI − 10 years of applications” of the International Journal of Biometeorology (Volume 65, Issue 9, September 2021), the book “Applications of the Universal Thermal Climate Index UTCI in biometeorology - Latest developments and case studies” (Krüger 2021a) and articles in different scientific journals. To avoid repetitions, only UTCI basics that are important for the objectives of this study are described below.

The UTCI was developed using the equivalent temperature approach (Schreier et al. 2013), i.e., the UTCI represents the equivalent air temperature (°C) of a reference environment that produces the same thermo-physiological responses of a standardised human-biometeorological reference person as the actual three-dimensional outdoor environment (Jendritzky et al. 2012). It is characterised by combined patterns of air temperature (*T*_*a*_), mean radiant temperature (*T*_*mrt*_) as measure for the radiant heat absorbed by humans, water vapour pressure (*VP*), and wind speed (*v*).

The reference environment was defined as (Błażejczyk et al. 2012, 2013; Pecelj et al. 2021): *T*_*a*_ = *T*_*mrt*_, relative humidity (*RH*): 50%, but the reference humidity was capped at *VP* = 20 hPa for *T*_*a*_ > 29 °C, and calm air, i.e., *v*_*10 m*_ = 0.5 m s^−1^ observed or simulated at 10 m above ground level (agl). As characteristics for the human-biometeorological reference person were set (Fiala et al. 2012): body surface area (Dubois area): 1.85 m^2^, body weight: 73.4 kg, body fat content: 14%, net metabolic heat production: 135 W m^−2^, and walking speed: 1.1 m s^−1^. These parameters are regarded as static in the UTCI determination, i.e., they cannot be changed.

The UTCI-Fiala multi-node model (Fiala et al. 2012) of human heat transfer and temperature regulation is the basic thermoregulation model to simulate human thermo-physiological responses to the actual outdoor meteorological conditions. It is combined with an adaptive outdoor clothing model (Havenith et al. 2012) that reflects a realistic insulation of the different human body segments. The insulation provided by clothing is behaviourally dependent on *T*_*a*_. The thermal and moisture resistances of the clothing are a function of *v* and the walking speed of the reference person. Thus, the overall insulation usually differs between the defined reference environment for calm air and the actual outdoor environment (Staiger et al. 2019).

Since the UTCI is an equivalent temperature, Bröde et al. (2012) described the UTCI deviation from *T*_*a*_, i.e., the offset, as a function of the actual values of *T*_*a*_, *T*_*mrt*_, *v* and *VP*:

1 $$\:UTCI\:=T_a+offset(T_a,\:T_{mrt},\:v,\:VP)$$

For simple and quick online UTCI calculations, the UTCI website (https://www.utci.org) provides a Fortran 90 subroutine that is based on very extensive test simulations for a variety of meteorological conditions in different climate zones. It approximates the UTCI offset to *T*_*a*_ by a 6th degree regression polynomial with *T*_*a*_, *T*_*mrt*_-*T*_*a*_, *VP*, and *v* as meteorological input variables. However, the application of this regression polynomial is limited to *v*_*10 m*_ values that are not lower than 0.5 m s^−1^ (Bröde et al. 2012; Coccolo et al. 2016; Schrijvers et al. 2016; Blażejczyk and Kuchcik 2021). Therefore, the UTCI cannot be calculated at very low *v*_*10 m*_ values (Fröhlich et al. 2019) as they are possible in the urban canopy layer (UCL), i.e., the outdoor air volume below the mean rooftops of buildings (Oke 1987; Barlow 2014; Mills 2014; Zajic et al. 2015; Wang et al. 2017; Pelliccioni et al. 2021; Kuttler and Weber 2023).

Due to the complexity of the 6th degree regression polynomial, Ren et al. (2023) and Liu et al. (2024) used an equation empirically derived by Błażejczyk (2011) to calculate the UTCI:

2 $$\:UTCI\:={3.21+0.872\times T}_a+0.2459\times T_{mrt}-2.5078\times v-0.0176\times RH$$

The comparatively high accuracy of UTCI results obtained by applying Eq. (2) is discussed in detail in Błażejczyk and Kunert (2011).

The operational procedure for calculating the UTCI is supplemented by a thermo-physiologically significant assignment of UTCI ranges to ten human thermal stress categories (Table S1 in the Supplementary material). This categorisation was derived from a comparison between (i) thermoregulatory variables and effector functions simulated by the UTCI-Fiala model (Fiala et al. 2012) and (ii) criteria from human thermo-physiology and ergonomics (Bröde et al. 2012; Bröde 2021). Several UTCI studies with regional climatic reference point out the need for calibrations of these UTCI ranges, taking into account physiological acclimatisation and psychological adaptation of the local population (Pantavou et al. 2014; Krüger et al. 2021c, d; Silva and Hirashima 2021; Potchter et al. 2022).

In the UTCI-Fiala multi-node model (Bröde et al. 2012), the convective heat exchange between sectors of the human-biometeorological reference person and the ambient air is described by an approach that includes a convection coefficient. It is determined as a function of body position, difference between the surface temperature of the body sector and the ambient *T*_*a*_, and local *v* at the representative height *z* (*v*_*z*_) of the respective body sector. Using the theoretically derived logarithmic law for the horizontally-averaged vertical wind speed profile (VWSP) under thermally neutral conditions (Oke 1987), *v*_*z*_ is calculated from *v*_*10 m*_ at the height of 10 m agl (*z*_*10 m*_):

3 $$\:v_z=v_{10\:m}\times\:\log(z/z_0)/\log(z_{10\:m}/z_0)$$

where *z*_*0*_ (m) is the roughness length in the upwind surrounding area. *z*_*0*_ = 0.01 m, which is assumed in the UTCI-Fiala multi-node model (Bröde 2021), represents the roughness length value for short-cut grassland (Wieringa 1992). Equation (3) is implemented in the UTCI basics and cannot be changed in whole or in part.

With regard to the announced universal applicability of the UTCI (Jendritzky et al. 2012), the UTCI basics do not contain any information on whether Eq. (3) actually meets this requirement. Results from micrometeorological studies on land use types with a larger vertical extent, such as cities or forests, already show that Eq. (3) can only be applied above these types, but not inside their pedestrian level (Oke 1987, 1988; Macdonald 2000).

For UTCI assessments of meteorological outdoor environments at different spatial scales, measured or simulated *v*_*10 m*_ values are not always available. This is particularly evident within the UCL. In this case, the UTCI developers recommend also using Eq. (3) to calculate *v*_*10 m*_ from *v* at lower heights (Bröde 2021).

Despite the human-biometeorological reference height (*z*_*h−b*_) of 1.1 m agl (Mayer 1993; Staiger et al. 2019), the meteorological input variables required to calculate the UTCI have different reference heights, such as 2 m agl for *T*_*a*_, *RH* or *VP*, and *T*_*mrt*_, and 10 m agl for *v*. This suggests that the development of the UTCI was primarily focused on products of the national weather services, i.e., standard ground-based data from observations and weather models (Fröhlich and Matzarakis 2020).

Meanwhile, the UTCI calculation is implemented in various micrometeorological application software (Bröde 2021), e.g., BioKlima 2.6 (Błażejczyk 2020), ENVI-met (Sinsel 2022), PALM model system 6.0 (Fröhlich and Matzarakis 2020), RayMan (Matzarakis et al. 2021a), and URBSIM (Schrijvers et al. 2016). For this reason, the UTCI has been widely applied around the world over the last decade (see literature review on UTCI applications by Krüger 2021b).

Unfortunately, a physically imprecise terminology can be found in UTCI studies (e.g., Pantavou et al. 2014; Battista et al. 2016; Chen et al. 2017; Li et al. 2020, 2022; Lian et al. 2020; Silva and Hirashima 2021; Peng et al. 2022). Current meteorological outdoor conditions or variables are often referred to as climatic or microclimatic conditions or variables, however without taking into account the characteristic time scale of climate of at least 30 years. Most UTCI calculations to date relate to much shorter time periods than those typical for climate, so that “climatic” or “microclimatic” should be consistently replaced by “meteorological” or “micrometeorological”. Accordingly, the name of the UTCI would make more sense without the inclusion of “climate”, e.g., only UTI (Universal Thermal Index) or UTAI (Universal Thermal Assessment Index).

### Air flow characteristics in the UCL

The air flow conditions in the UCL are influenced by complex roughness and thermal features due to varying building design, vegetation, and traffic structures. Applying experimental methods (field or wind tunnel studies) and model-based numerical simulations (e.g., large-eddy simulations), they have already been widely investigated for various reasons, e.g., analysis of natural ventilation in high-density cities or patterns of pedestrian-level air flow that affect local air quality and human thermal comfort (e.g., Zou et al. 2021; Chockalingam et al. 2023; Li et al. 2024a, b; Tang et al. 2024; Zeng et al. 2024). Due to the large number of results available for urban areas with heterogeneous roughness elements and land use types, only the air flow characteristics in the lower UCL that are relevant for the VWSP in the UTCI basics are briefly discussed below.

In principle, the three-dimensional air flow patterns within the UCL, especially at the street canyon scale, are heterogeneous and relatively complex (e.g., Macdonald 2000; Chew et al. 2017; Gronemeier et al. 2017; Lim et al. 2017; Wang et al. 2017; Buccolieri and Hang 2019; Barbano et al. 2021; Liu et al. 2023; Li et al. 2024c; Lu et al. 2024). The main reasons are their dependence on the air flow conditions above the roof level and the geometrical inhomogeneity in urban design. This includes varying aspect ratios (height-to-width) of street canyons, but also buildings, trees, vehicles, etc. that contribute to the deformation of the air flow patterns and generate turbulence and vortices of varying scales (Sato and Kusuka 2023). For a perpendicular inflow above the roof level, Oke (1988), following Hussain and Lee (1980), described the formation of specific air flow regimes in the UCL that depend on the depth of the urban street canyons. Hang et al. (2012) found for low *v* conditions that the thermal buoyancy force caused by shading and radiation trapping effects of buildings can have a lasting effect on instantaneous air flow patterns in the UCL. In this context, Gronemeier et al. (2017) pointed out the importance of unstable thermal stratification in analyses of the air flow in the heterogeneous UCL, which should be taken into account especially at low *v* conditions, as they are often typical in the UCL. In addition, He et al. (2022a, b) showed that the VWSPs in the UCL are highly variable due to the different sources and sinks of momentum and heat occurring there.

The summary of previous results from field studies, wind tunnel investigations and model-based numerical simulations on air flow patterns in the UCL including their three-dimensional visualisation reveals that the logarithmic law for the VWSP cannot be applied in the UCL (e.g., Buccolieri and Hang 2019; Theeuwes et al. 2019; Pelliccioni et al. 2021; Sun et al. 2024; Zeng et al. 2024). The main reasons for the inapplicability of Eq. (3) in the UCL are: (i) it uses a *z*_*0*_ value only typical for short-cut grassland and not for other urban land use types (Oke 1987); (ii) it only refers to neutral atmospheric stability, which, however, is not always the norm within urban outdoor spaces, particularly at locations with low wind speed, higher solar radiant flux densities and dense urban fabric such as in Hong Kong (Gronemeier et al. 2017); (iii) it does not take into account the different generic airflow patterns within spatially heterogeneous urban geometries (Oke 1988). The *v* conditions at specific locations in an urban street canyon can be analysed using CFD/LES simulations or a combination of the approaches by Macdonald (2000) for the flow along-canyon flow and Yamartino and Wiegand (1986) for the cross-canyon vortex.

### Objectives of this study

The thermo-physiological basics of the UTCI are considered to be more precise (Weihs et al. 2012; Staiger et al. 2019) than those of similar thermal assessment indices (Potchter et al. 2018; Staiger et al. 2019), e.g., physiologically equivalent temperature (PET; Mayer and Höppe 1987), perceived temperature (PT; Staiger et al. 2012), and modified physiologically equivalent temperature (mPET; Chen and Matzarakis 2018), which were also derived from the human heat balance. Nevertheless, the accuracy of UTCI results also depends on the accuracy of the meteorological input variables (Schreier et al. 2013; Bröde 2021). Weihs et al. (2012) concluded that usual inaccuracies of the four meteorological input variables lead to total UTCI inaccuracies of up to 6 K at most, i.e., one step in the UTCI assessment categorisation (Table S1). In particular, the UTCI values react very sensitively to changes in *T*_*mrt*_ and *v* (Błażejczyk et al. 2012; Bröde et al. 2012; Provençal et al. 2016). Related to inaccuracies in numerical simulations of *T*_*mrt*_, Weihs et al. (2012) and Schreier et al. (2013) found for clear-sky weather that the UTCI inaccuracies are not higher than 2 K. However, if the information on cloud cover is incorrect, the UTCI inaccuracies can also be up to 6 K.

Schreier et al. (2013) and Geletič et al. (2021a) already pointed out UTCI inaccuracies caused by the impact of *v*, but they did not carry out any corresponding investigations themselves. Rather, they argued that this problem is a challenging and interesting topic for future studies looking at the accuracy of UTCI values. In the context of a comparative analysis of the UTCI pros and UTCI cons, Park et al. (2014) emphasised the need for suitable and accurate *v* values because they are of great importance for UTCI applications in the UCL. Fröhlich and Matzarakis (2020) have also critically analysed the role of the VWSP approach in the UTCI basics and concluded that it is inappropriate for UTCI calculations in the UCL.

In principle, the UTCI cannot be applied at the urban pedestrian level because the approach for the VWSP in the UTCI basics is not consistent with micrometeorological principles and existing knowledge about air flow in the UCL. If these concerns are ignored and the UTCI is still applied at the urban pedestrian level, there are two further issues resulting in UTCI inaccuracies: (i) the exclusive reference to neutral atmospheric stability, i.e., other thermal stratifications in the UCL are not considered, and (ii) the unchangeable *z*_*0*_ value for short-cut grassland in the UTCI basics, which does not represent urban land use types. Assuming a neutrally stratified UCL, this case study only addresses the *z*_*0*_ issue, primarily for Central European summer conditions. Its objectives are: (i) to analyse the influence of *z*_*0*_ on *v*_*h−b*_ and *v*_*10 m*_ applying Eq. (3) for the VWSP and (ii) to estimate the resulting inaccuracies of UTCI values that may arise at the urban pedestrian level in three different climate zones caused by using *z*_*0*_ for short-cut grassland instead of *z*_*0*_ for a typical urban land use.

## Methodology

Among the meteorological input variables for UTCI calculations at the urban pedestrian level, *T*_*a*_ and *VP* or *RH* are generally considered to be those that can be determined comparatively easily in field studies or by numerical simulations (Nazarian 2022; Martilli et al. 2024). They often have only very slight vertical gradients in the UCL (Ketterer et al. 2017), mostly within measurement or simulation accuracies (Coutts et al. 2016).

However, the determination of *T*_*mrt*_ and *v* in the immediate vicinity of pedestrians has certain shortcomings in the heterogeneous UCL (Pecelj et al. 2021), albeit of varying extent. Since *T*_*mrt*_ cannot be measured directly at *z*_*h−b*_, methods have been developed to make *T*_*mrt*_ available indirectly. They include (i) experimental approaches, e.g., the globe thermometer method proposed by Thorsson et al. (2007) as currently the most common method (e.g., Kántor and Unger 2011; Kántor et al. 2015; Liu et al. 2022) or the more complex six-directional method (e.g., Thorsson et al. 2007; Mayer et al. 2008; Holst and Mayer 2011; Lee et al. 2013, 2022; Middel and Krayenhoff 2019), and (ii) numerical simulations (Lam et al. 2021b) with area-related models that are suitable for applications in the UCL, e.g., ENVI-met (e.g., Sinsel et al. 2022; Lee et al. 2023), PALM-4U (Fröhlich and Matzarakis 2020), and SOLWEIG (Lindberg et al. 2016) or models for only individual locations such as RayMan (e.g., Lee and Mayer 2016). These methods are very often used for issues in urban human-biometeorology (e.g., Kántor and Unger 2011; Mayer et al. 2008; Gal and Kántor 2020; Jin et al. 2021; Lee et al. 2022; Sinsel et al. 2022; Jia et al. 2024). Irrespective of the accuracy achieved for *T*_*mrt*_ (e.g., Crank et al. 2020; Gal and Kántor 2020; Jin et al. 2021; Fang et al. 2023; Wallenberg et al. 2023), the problems in determining *T*_*mrt*_ can superficially be seen as solvable, even if with varying degrees of effort.

With regard to the *v* issue, the value of *z*_*0*_ = 0.01 m in Eq. (3) is clearly too low for UTCI applications in the UCL (Maras et al. 2016) because this layer is characterised by different building structures and an irregular heterogeneity of surface features (e.g., Chen et al. 2017; Geletič et al. 2021a). In order to determine the influence of different *z*_*0*_ values on *v*_*z*_ (z < z_10 m_) when applying Eq. (3), simulations were performed for two *v*_*10 m*_ values (1.0 m s^−1^ and 2.0 m s^−1^). They were determined on the basis of human-biometeorological field studies (Mayer et al. 2008; Holst and Mayer 2011) conducted from 2007 to 2009 on Central European summer days at 89 different sites within the city of Freiburg (southwest Germany; humid temperate climate (Cfb) according to the updated Köppen-Geiger climate classification by Kottek et al. (2006)).

By reformulating Eq. (3) into

4 $$\:v_z=v_{h-b}\times\:\log(z/z_0)/\log(z_{h-b}/z_0)$$

the *z*_*0*_ influence on *v*_*z*_ (z_h−b_ < z ≤ z_10 m_) was exemplarily analysed for two *v*_*h−b*_ values (0.6 m s^−1^ and 0.4 m s^−1^). They were also set on the basis of the results of these field studies and were considered representative of *v*_*h−b*_ under the meteorological conditions during the field studies.

Also related to these micrometeorological conditions in Freiburg at midday and at midnight, the maximum UTCI inaccuracies dependent on *T*_*a*_ were determined as UTCI differences (ΔUTCI) between the UTCI for *z*_*0*_ = 0.01 m (unchangeable default value in the UTCI basics) and the UTCI for *z*_*0*_ = 0.80 m (roughness length for a typical urban land use according to Maras et al. (2016)). The UTCI values were computed using the UTCI calculator on the UTCI website (https://www.utci.org/utci_calc.php). In order to isolate the influence of only *z*_*0*_ on the UTCI, *RH* = 50% was assumed in both periods. According to the findings of the aforementioned field studies in Freiburg, in which *T*_*mrt*_ was determined using the complex and therefore relatively accurate six-directional method, *T*_*mrt*_ = 2·*T*_*a*_ was set at midday and *T*_*mrt*_ = *T*_*a*_ at midnight. Applying the logarithmic law for the VWSP under neutral atmospheric stability, *v*_*10 m*_ was calculated from the two previously mentioned *v*_*h−b*_ values (0.4 m s^−1^ and 0.6 m s^−1^) according to Eq. (4).

In order to compare the ΔUTCI results obtained for the humid temperate climate of Freiburg (Cfb) with similar results for other climate zones, additional ΔUTCI determinations were carried out for the hot and dry desert climate of Ghardaia, Algeria (BWh), and the humid subtropical climate of Hong Kong (Cwa), each based on TMY datasets (https://climate.onebuilding.org/) for the meteorological input variables. For these supplementary ΔUTCI calculations, the assumptions regarding *v*_*h−b*_ and *T*_*mrt*_ that had been made for the UTCI simulations in Freiburg were retained, while the *RH* values were adjusted to the respective climate. For Ghardaia, *RH* was set at 18% at midday and 20% at midnight, and for Hong Kong, *RH* was set at 77% at midday and 85% at midnight.

## Results

### Influence of ***z***_***0***_on the vertical wind speed profile in the lower UCL

Taking into account higher *z*_*0*_ values, which are more typical of urban land use types than *z*_*0*_ = 0.01 m, and assuming a constant *v*_*10 m*_, the VWSP curvatures in the layer between *z*_*h−b*_ and *z*_*10 m*_ become more pronounced with increasing *z*_*0*_ as they are controlled according to Eq. (3) by log(*z*/z_0_)/log(*z*_*10 m*_/*z*_*0*_). This pattern is shown in Fig. S1 for *v*_*10 m*_ = 1.0 m s^−1^ and Fig. 2 for *v*_*10 m*_ = 2.0 m s^−1^ and resulting in significantly lower *v*_*h−b*_ values with higher *z*_*0*_ (Table S2, Fig. S2). If, for example, *z*_*0*_ is increased from 0.01 m to 0.50 m, *v*_*h−b*_ is reduced from 0.68 m s^−1^ to 0.26 m s^−1^, i.e., by 0.42 m s^−1^, for *v*_*10 m*_ = 1.0 m s^−1^ and from 1.36 m s^−1^ to 0.52 m s^−1^, i.e., by 0.84 m s^−1^, for *v*_*10 m*_ = 2.0 m s^−1^. However, if *z*_*0*_ is increased from 0.01 m to 0.80 m, *v*_*h−b*_ is reduced by 0.55 m s^−1^ for *v*_*10 m*_ = 1.0 m s^−1^ and by 1.10 m s^−1^ for *v*_*10 m*_ = 2.0 m s^−1^.

**Fig. 1:**
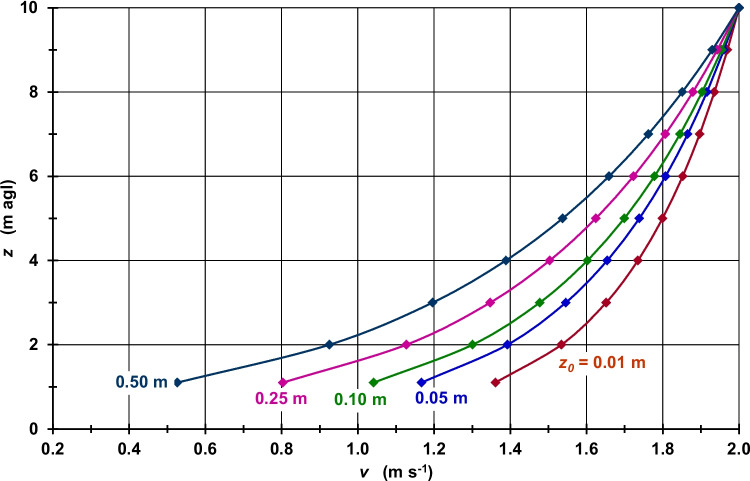
Wind speed (*v*) as a function of height (*z*) and roughness length (*z*_*0*_); *v*_*10 m*_ = 2.0 m s^−1^; neutral atmospheric stability

**Fig. 2:**
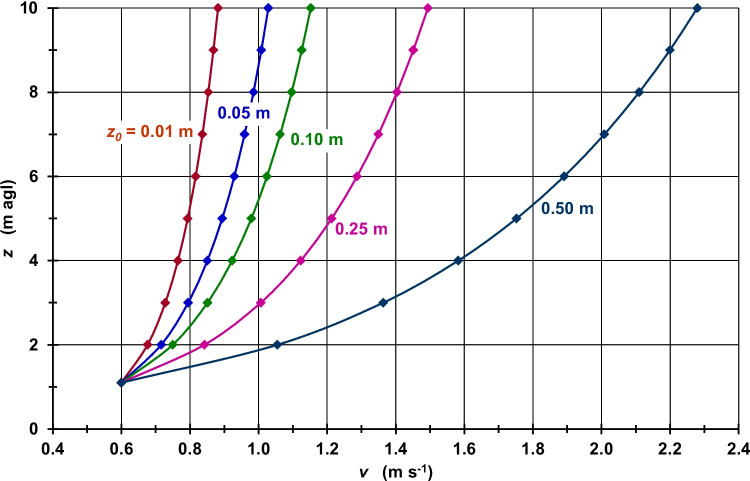
Wind speed (*v*) as a function of height (*z*) and roughness length (*z*_*0*_); *v*_*h−b*_ = 0.6 m s^−1^; neutral atmospheric stability

Referring to Eq. (4), Figs. S3 (for *v*_*h−b*_ = 0.4 m s^−1^) and 2 (for *v*_*h−b*_ = 0.6 m s^−1^) indicate higher *v*_*z*_ values from *z*_*h−b*_ to *z*_*10 m*_ caused by a *z*_*0*_ increase from 0.01 m to 0.50 m (see also Fig. S2). This increase in *z*_*0*_ causes an increase in *v*_*10 m*_ from 0.59 m s^−1^ to 1.52 m s^−1^, i.e., by 0.93 m s^−1^, for *v*_*h−b*_ = 0.4 m s^−1^ and from 0.88 m s^−1^ to 2.28 m s^−1^, i.e., by 1.40 m s^−1^, for *v*_*h−b*_ = 0.6 m s^−1^ (Table S3). An increase in *z*_*0*_ from 0.01 m to 0.80 m leads to *v*_*10 m*_ values that are 2.58 m s^−1^ higher for *v*_*h−b*_ = 0.4 m s^−1^ and 3.88 m s^−1^ higher for *v*_*h−b*_ = 0.6 m s^−1^. Similar to Figs. 1 and S2, the VWSP curvatures in the layer between *z*_*h−b*_ and *z*_*10 m*_ also become more pronounced with increasing *z*_*0*_ (Figs. 2 and S3) because they are now controlled according to Eq. (4) by log(*z*/z_0_)/log(*z*_*h−b*_/*z*_*0*_).

### Maximum UTCI inaccuracies at the urban pedestrian level due to an increase of ***z***_***0***_

**Fig. 3:**
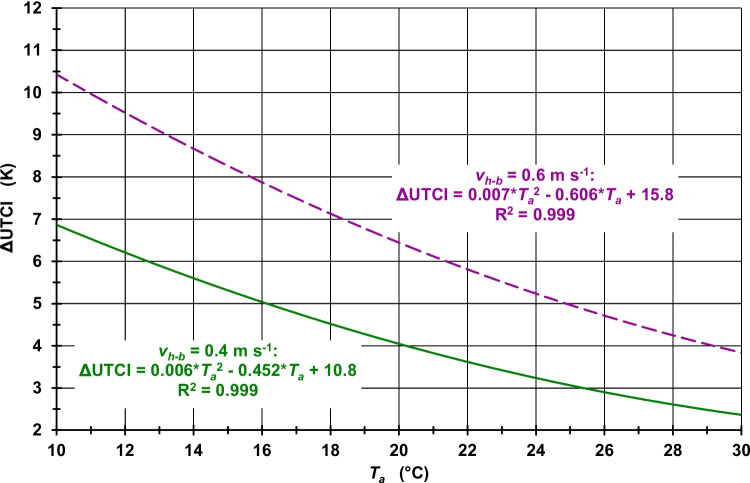
UTCI differences (ΔUTCI) between *v*_*10 m*_, calculated according to the logarithmic law for the VWSP from *v*_*h−b*_ and *z*_*0*_ = 0.01 m, and *v*_*10 m*_, calculated by the same method but for *z*_*0*_ = 0.80 m, for two *v*_*h−b*_ values; neutral atmospheric stability; assumption: *T*_*mrt*_ = 2·*T*_*a*_ and *RH* = 50% (Freiburg, humid temperate climate (Cfb), July, at midday); R^2^: coefficient of determination

**Fig. 4:**
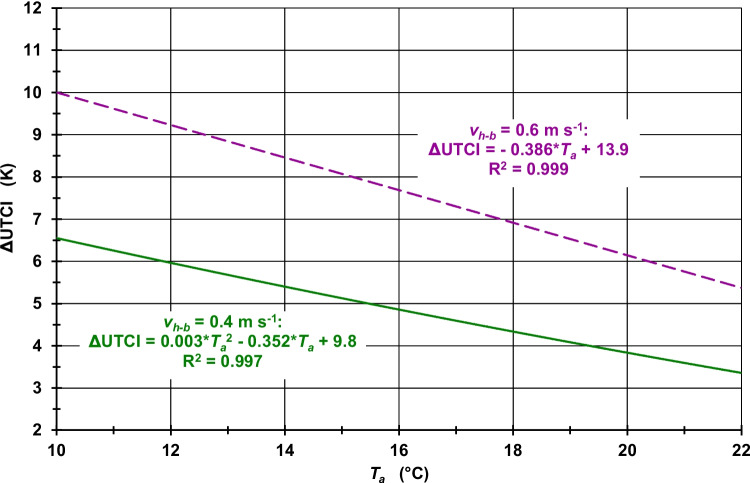
UTCI differences (ΔUTCI) between *v*_*10 m*_, calculated according to the logarithmic law for the VWSP from *v*_*h−b*_ and *z*_*0*_ = 0.01 m, and *v*_*10 m*_, calculated by the same method but for *z*_*0*_ = 0.80 m, for two *v*_*h−b*_ values; neutral atmospheric stability; assumption: *T*_*mrt*_ = *T*_*a*_ and *RH* = 50% (Freiburg, humid temperate climate (Cfb), July, at midday); R^2^: coefficient of determination

The increase in *v*_*10 m*_ due to the increase in *z*_*0*_ from 0.01 m to 0.80 m results in a decrease in the UTCI. Therefore ΔUTCI is greater than 0 K in both study periods. Although the UTCI values increase with higher *T*_*a*_ values, which also implies higher *T*_*mrt*_ values, the maximum UTCI inaccuracies in terms of ΔUTCI values decrease with increasing *T*_*a*_, both at midday (Fig. 3 for Freiburg, S4 for Ghardaia, and S6 for Hong Kong) and at midnight (Fig. 4 for Freiburg, S5 for Ghardaia, and S7 for Hong Kong). The main reason for this pattern is the increasing influence of higher *T*_*a*_ values in the UTCI calculations, while *v*_*10 m*_ only depends on *v*_*h−b*_ and *z*_*0*_. As this *z*_*0*_-dependent increase in *v*_*10 m*_ is 1.59 m s^−1^ higher for *v*_*h−b*_ = 0.6 m s^−1^ than for *v*_*h−b*_ = 0.4 m s^−1^ (Table S3), the ΔUTCI values are higher for *v*_*h−b*_ = 0.6 m s^−1^ as compared to *v*_*h−b*_ = 0.4 m s^−1^, each for the same *T*_*a*_ values. Due to the different impacts of *T*_*a*_ changes in contrast to the *v*_*10 m*_ conditions on the UTCI, the ΔUTCI values for *v*_*h−b*_ = 0.4 m s^−1^ and *v*_*h−b*_ = 0.6 m s^−1^ are approaching with higher *T*_*a*_ values. The described pattern of ΔUTCI values ​​is also reflected quantitatively in Table 1, which contains ΔUTCI values ​​depending on *v*_*h−b*_ and two selected *T*_*a*_ values ​​at midday and at midnight.

**Table 1:** ΔUTCI values dependent on *v*_*h−b*_ and *T*_*a*_ for three cities in different climate zones according to the updated Köppen-Geiger climate classification (Kottek et al. 2006), typical summer days in July at midday and at midnight

period	v_h−b_ (m s^−1^)	T_a_ (°C)	ΔUTCI (K)
Freiburg (Germany, humid temperate climate, Cfb)			
at midday	0.4	24.0	3.2
	0.6	24.0	5.2
	0.4	30.0	2.4
	0.6	30.0	3.9
at midnight	0.4	18.0	4.4
	0.6	18.0	6.9
	0.4	22.0	3.3
	0.6	22.0	5.4
Ghardaia (Algeria, hot and dry desert climate, BWh)			
at midday	0.4	30.0	1.6
	0.6	30.0	2.9
	0.4	36.0	0.8
	0.6	36.0	1.7
at midnight	0.4	24.0	2.8
	0.6	24.0	4.7
	0.4	28.0	1.8
	0.6	28.0	3.3
Hong Kong (humid subtropical climate, Cwa)			
at midday	0.4	30.0	2.7
	0.6	30.0	4.1
	0.4	36.0	2.4
	0.6	36.0	3.4
at midnight	0.4	24.0	3.1
	0.6	24.0	4.9
	0.4	28.0	2.5
	0.6	28.0	3.8

The meteorological conditions, which are typical for the humid temperate climate of Freiburg, result in ΔUTCI values between 2.4 K and 5.2 K at midday and 3.3 K and 6.9 K at midnight (Table 1). Since higher *T*_*a*_ but lower *RH* values were assumed for Ghardaia than for Freiburg due to its hot and dry desert climate, the ΔUTCI values were between 0.8 K and 2.9 K at midday and 1.8 K and 4.7 K at midnight. Compared to Ghardaia, the same *T*_*a*_ but significantly higher *RH* values ​​were assumed for Hong Kong (humid subtropical climate) resulting in ΔUTCI values between 2.4 K and 4.1 K at midday and 2.5 K and 4.9 K at midnight. Since in Table 1 the *T*_*a*_ reference values ​​for Ghardaia and Hong Kong are identical, it can be easily recognized that (i) the higher *RH* values ​​in Hong Kong lead to higher ΔUTCI values ​​in contrast to lower ΔUTCI values ​​in dry Ghardaia, and (ii) the differences in the ΔUTCI values between Hong Kong and Ghardaia increase with higher *T*_*a*_.

In the case of calculating *v*_*h−b*_ from the input variable *v*_*10 m*_ by applying Eq. (3), the increase in *z*_*0*_ from 0.01 m to higher values results in a *v*_*10 m*_-dependent reduction of *v*_*h−b*_, as shown in Figs. 1 and S2. Since *z*_*0*_ = 0.01 m is fixed in the UTCI basics, a realistic *z*_*0*_ for the UCL would lead to an increase in the UTCI, i.e., ΔUTCI would be negative. However, it is impossible to estimate its extent precisely because it cannot be determined using the tools freely available for calculating the UTCI.

Overall, the use of *z*_*0*_ = 0.01 m instead of *z*_*0*_ = 0.80 m results in an overestimation of the UTCI in the case that *v*_*10 m*_ is calculated from *v*_*h−b*_ and an underestimation of the UTCI in the case that *v*_*h−b*_ is calculated from *v*_*10 m*_. Due to the *v* differences in the respective target heights caused by the two differing *z*_*0*_ values, it can be assumed that the amount of this UTCI underestimation is lower than that of this UTCI overestimation. Therefore, if both cases occur together, the maximum UTCI inaccuracies, whose amounts are additionally modified by the current *T*_*a*_, *T*_*mrt*_, *RH* and *v*_*h−b*_ or *v*_*10 m*_ conditions, are at least as large as the UTCI inaccuracies due inaccuracies in simulated *T*_*mrt*_ values (Weihs et al. 2012; Schreier et al. 2013).

## Discussion

In the last decade, the UTCI has been increasingly applied to assess the meteorological outdoor environment at different urban locations under a human-biometeorologically significant perspective (e.g., literature review on UTCI applications by Krüger 2021b; Jiménez and de Adana 2024), but mostly without checking to what extent and whether UTCI is applicable for thermo-physiologically significant assessments at the pedestrian level within the complex and heterogeneous UCL at all (e.g.Bröde et al. 2013; Battista et al. 2016; Coutts et al. 2016; Provençal et al. 2016; Krüger 2017; Cheung and Jim 2018; Pantavou et al. 2018; Lee et al. 2020; Li et al. 2020; Silva and Hirashima 2021; Krüger et al. 2021c; Lam et al. 2021a; Peng et al. 2022; Bröde and Kampmann 2023; Diz-Mellado et al. 2023; Yang et al. 2023; Yi et al. 2023; Briegel et al. 2024; Ding et al. 2024; Jing et al. 2024; Zhou et al. 2025). The decisive factor is not so much the more well-founded thermo-physiological basics of the UTCI compared to those of PET, mPET or PT (e.g., Park et al. 2014; Coccolo et al. 2016; Lin et al. 2019; Pecelj et al. 2021), but rather the free availability of various tools for UTCI calculations (e.g.Geletič et al. 2021a, b, 2023; Matzarakis et al. 2021a, b; Katavoutas et al. 2022; Nie et al. 2022; Savić et al. 2022; Speak and Salbitano 2022; Hao et al. 2023; Heidari et al. 2024; Ouyang et al. 2024; Tomasi et al. 2024).

Almost all previous UTCI studies at the urban pedestrian level do not address the fact that the logarithmic law for the VWSP under thermally neutral conditions (Eqs. (3) and (4)) generally cannot be applied in the UCL if micrometeorological principles are to be kept. The issues associated with the VWSP approach also include the shortcoming of using the *z*_*0*_ value (0.01 m) for short-cut grassland. With the exception of the study by Maras et al. (2016), previous UTCI analyses in the UCL did not discuss the usefulness of the *z*_*0*_ value of 0.01 m for the pedestrian level in the UCL. The study by Maras et al. (2016) is the only experimental investigation in the UCL to date applying a *z*_*0*_ value (0.80 m), which is significantly more representative of the UCL than *z*_*0*_ = 0.01 m.

In this context, the simulations reported here were carried out to analyse the impacts of *z*_*0*_ values ​​between 0.01 m and 0.80 m on the VWSP in the lower UCL, in particular on *v*_*h−b*_ (Eq. (3)) and *v*_*10 m*_ (Eq. (4)). They were supplemented by calculations of the resulting maximum UTCI inaccuracies at the pedestrian level in three cities in different climate zones, but only under typical meteorological conditions in the summer month of July. The simulation results on the impacts of *z*_*0*_ were presented as ranges for the target variables *v*_*h−b*_, *v*_*10 m*_, and ΔUTCI. They reached a quantitative level that cannot be classified as negligible. Since typical meteorological conditions in July in three cities located in different climate zones were taken into account in this study, a climate zone-specific influence on UTCI could be identified in addition to the *z*_*0*_ influence. Unfortunately, all results obtained in this simulation study cannot be evaluated in terms of their general resilience because no similar case studies, e.g., about maximum UTCI inaccuracies under further climatic or atmospheric stability conditions, are known to date.

Instead of the logarithmic law, the empirically derived simple power law for the horizontally-averaged VWSP according to Deacon (Oke 1987)

5 $$\:v_z=v_{ref}\times\left(z/z_{ref}\:\right)^{\alpha\:}$$

is sometimes used in human-biometeorological assessments at the urban pedestrian level (e.g., Fröhlich and Matzarakis 2013; Ketterer and Matzarakis 2014; Martinelli et al. 2015; Nastos and Polychroni 2016; Lian et al. 2020; Matzarakis et al. 2021b; Pecelj et al. 2021; He et al. 2022a; Cárdenas-Jirón et al. 2023). The power law exponent *α* depends on local conditions such as height, real surface roughness of the upwind area and atmospheric stability (Nakajima et al. 2020). Commonly, only a single value of *α*, often between 0.21 and 0.50 (Lim et al. 2017; He et al. 2022a, b), is frequently taken from tables as an average value for individual urban land use types, i.e., *α* doesn’t vary with height and atmospheric stability or directly consider the current surface roughness (Kent et al. 2018). Sometimes, *α* is also determined by the empirical approach (Fröhlich and Matzarakis 2013; Ketterer et al. 2017):

6 $$\:\alpha\:={0.12\times z}_0+0.18$$

However, the shortcomings described when using the logarithmic law for the VWSP in UTCI calculations at the urban pedestrian level do not change if the power law is applied instead. As clearly shown by Lim et al. (2017), *α* is not a constant, since the upwind area relevant for *z*_*0*_ depends on the often uneven wind direction in the heterogeneous UCL and can therefore vary significantly in extent and morphological structure. Lee and Mayer (2016) had already pointed out this issue when validating *T*_*mrt*_ simulations by the RayMan software package. It also uses the Eqs. (5) and (6), which were identified as a cause for inaccurate *T*_*mrt*_ simulations by RayMan.

According to Pelliccioni et al. (2021) and He et al. (2022b), both the logarithmic and the power law are more applicable to homogeneous areas under neutral atmospheric stability, but with the limitation that the power law is in principle unsuitable to describe the VWSP at lower levels in the UCL (Drew et al. 2013; Li et al. 2024a). Due to the heterogeneous surface roughness and diverse land use types in urban areas, which lead to different frictional resistance and varying sources and sinks of heat (Wang et al. 2023), the spatiotemporal variability of the VWSP in the UCL should be considered in a more appropriate VWSP approach than in the current UTCI basics.

A comparatively simple way to eliminate the previously discussed shortcomings with regard to the VWSP approach and *z*_*0*_ in the UTCI basics would be if all meteorological input variables for UTCI calculations at the urban pedestrian level, i.e., not only *T*_*a*_, *T*_*mrt*_ and *VP* or *RH* but also *v*, originate from the human-biometeorological reference height (*z*_*h−b*_), as is the case in calculations of comparable thermo-physiological assessment indices such as PET, mPET or PT. This methodological variant in an update of the UTCI basics does not require *z*_*0*_ values and is not complex, because *v*_*h−b*_ frequently represents a direct result from experimental investigations or numerical simulations in the UCL. With this procedure, the local variability of *v*_*h−b*_ within different urban morphologies and canyon cross-sections could be directly considered in UTCI calculations.

## Conclusions

The UTCI developers have made the claim that the UTCI should be universally applicable in all climates, all seasons, and in spatial and temporal dimensions from the micro to the macro scale (Błażejczyk et al. 2013; Bröde et al. 2013). This requirement cannot be met for UTCI applications at the urban pedestrian level due to the VWSP approach, i.e., the logarithmic law for the VWSP under thermally neutral conditions including the *z*_*0*_ value for short-cut grassland, which is invariably implemented in the UTCI basics. This VWSP approach is only applicable to a homogeneous and neutrally stratified boundary layer and not to a heterogeneous and convective UCL (He et al. 2022a). If precise micrometeorological principles are not to be disregarded in the UCL, it is recommended that the UTCI not be used for thermo-physiologically significant assessments of outdoor environments in the UCL, regardless of how large the resulting UTCI inaccuracies would be. If the UTCI is nevertheless applied in the lower UCL, which is frequently done (see citations in this study), inaccuracies in the UTCI results are to be expected due to the aforementioned shortcomings in the VWSP approach. Only related to the *z*_*0*_ issue, the ΔUTCI values in this study showed climate zone-dependent ranges already reaching up to 7 K.

Unless there is an update of the UTCI basics that eliminates the shortcomings related to the current VWSP approach and the associated *z*_*0*_ value for UTCI applications within the UCL, other thermo-physiologically significant assessment indices, e.g., PET, mPET or PT, can be applied to assess the thermal outdoor conditions at the heterogeneous urban pedestrian level. Although their thermo-physiological basics are not as well-founded as those of the UTCI, they have the advantage of using localised *v*_*h−b*_ values ​​as input variables. In this context, Brecht et al. (2020) concluded that the UTCI is suited for operational meteorological data and applications at spatial scales from city quarters onwards because it requires *v*_*10 m*_, whereas the other thermo-physiological assessment indices that directly use *v*_*h−b*_ are more suited for building- and street-resolving calculations. This differentiated evaluation is acceptable.

In addition to cities, forests also represent a land use type with a comparatively large vertical extent. Whereas cities form an UCL, the stand space in forests has micrometeorological characteristics that are quite similar to those within the UCL. Due to comparable VWSP issues, the UTCI can also not be recommended for human-biometeorological assessments of thermal conditions within forest stands.

## Supplementary information

Below is the link to the electronic supplementary material.

ESM 1 (DOCX 310 KB)

## Data Availability

The datasets generated during and/or analyzed during the current study are available from the corresponding author on reasonable request.

## Nomenclature

α: power law exponent
agl: above ground level
PET: °C, physiologically equivalent temperature
mPET: °C, modified physiologically equivalent temperature
PT: °C, perceived temperature
*RH*: %, relative humidity
R^2^: coefficient of determination
*T*_*a*_: °C, air temperature
*T*_*mrt*_: °C, mean radiant temperature
UCL: urban canopy layer
UTCI: °C, Universal Thermal Climate Index
*v*: m s^-1^, wind speed
*v*_*h-b*_: m s^-1^, wind speed at z_*h-b*_
*v*_*h-b,z0*_: m s^-1^, wind speed at z_*h-b*_ dependent on z_*0*_
*v*_*h-b,0.01 m*_: m s^-1^, wind speed at z_*h-b*_ for z_*0*_ = 0.01 m
v_z_: m s^-1^, wind speed at the height z
*v*_*10 m*_: m s^-1^, wind speed at z_*10 m*_
*v*_*10 m,z0*_: m s^-1^, wind speed at z_*10 m*_ dependent on z_*0*_
*v*_*10 m,0.01 m*_: m s^-1^, wind speed at z_*10 m*_ for z_*0*_ = 0.01 m
*VP*: hPa, water vapour pressure
VWSP: vertical wind speed profile
*z*: m, height agl
*z*_*h-b*_: m, human-biometeorological reference height (1.1 m agl)
*z*_*10 m*_: m, height of 10 m agl
*z*_*0*_: m, roughness length
