# Correction to: Approach for the vertical wind speed profile implemented in the UTCI basics blocks UTCI applications at the urban pedestrian level

**DOI:** 10.1007/s00484-025-02915-6

**Published:** 2025-04-14

**Authors:** Hyunjung Lee, Sookuk Park, Helmut Mayer

**Affiliations:** 1 Department of Environmental Engineering, College of Ocean Science, Jeju National University, Jeju, Republic of Korea; 2 Laboratory of Landscape Architecture, Department of Horticultural Science, College of Applied Life Science, Jeju National University, Jeju, Republic of Korea; 3 Chair of Environmental Meteorology, Albert-Ludwigs-University of Freiburg, Freiburg, Germany

**Correction to: International Journal of Biometeorology (2024) 69:567–580**

10.1007/s00484-024-02835-x

The article “Approach for the vertical wind speed profile implemented in the UTCI basics blocks UTCI applications at the urban pedestrian level”, written by Lee, H., Park, S. & Mayer, H., was originally published under exclusive license to Springer Science + Business Media, LLC, part of Springer Nature. As a result of the subsequent decision to publish the article under the open access model, the article’s copyright notice was changed on 20 March 2025 to © The Author(s) 2025 and the article is now distributed under a Creative Commons Attribution 4.0 International License, which permits use, sharing, adaptation, distribution and reproduction in any medium or format, as long as you give appropriate credit to the original author(s) and the source, provide a link to the Creative Commons licence, and indicate if changes were made. The images or other third party material in this article are included in the article’s Creative Commons licence, unless indicated otherwise in a credit line to the material. If material is not included in the article’s Creative Commons licence and your intended use is not permitted by statutory regulation or exceeds the permitted use, you will need to obtain permission directly from the copyright holder. To view a copy of this licence, visit http://creativecommons.org/licenses/by/4.0. Open access funding enabled and organized by Projekt DEAL.

