# Supplementary material for: Approach for the vertical wind speed profile implemented in the UTCI basics blocks UTCI applications at the urban pedestrian level
Source: Int J Biometeorol. 2024 Dec 3;69(3):567–80. doi: 10.1007/s00484-024-02835-x (PMC11976762; doi:10.1007/s00484-024-02835-x)
Supplement: Supplementary file 1 — (DOCX 310 KB) [file 484_2024_2835_MOESM1_ESM.docx]

Supplementary material to the article

**Approach for the vertical wind speed profile implemented in the UTCI basics blocks UTCI applications at the urban pedestrian level**

**Hyunjung Lee^1^ • Sookuk Park^2^ • Helmut Mayer^3^**

Hyunjung Lee

hyunjunglee@jejunu.ac.kr

Sookuk Park

sooland@jejunu.ac.kr

🖂 Helmut Mayer

helmut.mayer@meteo.uni-freiburg.de

1 Department of Environmental Engineering, College of Ocean Science, Jeju National University, Republic of Korea

2 Laboratory of Landscape Architecture, Department of Horticultural Science, College of Applied Life Science, Jeju National University, Republic of Korea

3 Chair of Environmental Meteorology, Albert-Ludwigs-University of Freiburg, Germany

c

**Table of contents**

**Page S3:**

**Table S1** Assignment of UTCI ranges to human thermal stress categories (according to Bröde et al. 2012)

**Table S2** Results for log(*z_h-b_*)⁄(*z_0_*))⁄(log(*z_10 m_*)⁄*z_0_*), *v_h-b,z0_* (for two *v_10 m_* values), and
*v*_h-b,z0_/*v*_h-b,0.01 m_, each dependent on *z_0_*

**Page S4:**

**Table S3** Results for log(*z*_10 m_)⁄(*z_0_*))⁄(log(*z_h-b_*)⁄*z_0_*), *v_10 m,z0_* (for two *v_h-b_* values), and
*v*_10 m,z0_/*v*_10 m,0.01 m_, each dependent on *z_0_*

**Fig. S1** Wind speed (*v*) as a function of height (*z*) and roughness length (*z_0_*); *v_10 m_* = 1.0 m s^-1^; neutral atmospheric stability

**Page S5**

**Fig. S2** *v_h-b,z0_*/*v_h-b,0.01 m_* and *v_10 m,z0_*/*v_10 m,0.01 m_* values dependent on the roughness length (*z_0_*); neutral atmospheric stability

**Fig. S3** Wind speed (*v*) as a function of height (*z*) and roughness length (*z_0_*); *v_h-b_* = 0.4 m s^-1^; neutral atmospheric stability

**Page S6**

**Fig. S4** UTCI differences (ΔUTCI) between *v_10 m_*, calculated according to the logarithmic law for the VWSP from *v_h-b_* and *z_0_* = 0.01 m, and *v_10 m_*, calculated by the same method but for *z_0_* = 0.80 m, for two *v_h-b_* values; neutral atmospheric stability; assumption: *T_mrt_* = 2·*T_a_* and *RH* = 18% (Ghardaia, Algeria, hot and dry desert climate (BWh), July, at midday); R^2^: coefficient of determination

**Fig. S5** UTCI differences (ΔUTCI) between *v_10 m_*, calculated according to the logarithmic law for the VWSP from *v_h-b_* and *z_0_* = 0.01 m, and *v_10 m_*, calculated by the same method but for *z_0_* = 0.80 m, for two *v_h-b_* values; neutral atmospheric stability; assumption: *T_mrt_* = *T_a_* and *RH* = 20% (Ghardaia, Algeria, hot and dry desert climate (BWh), July, at midnight); R^2^: coefficient of determination

**Page S7**

**Fig. S6** UTCI differences (ΔUTCI) between *v_10 m_*, calculated according to the logarithmic law for the VWSP from *v_h-b_* and *z_0_* = 0.01 m, and *v_10 m_*, calculated by the same method but for *z_0_* = 0.80 m, for two *v_h-b_* values; neutral atmospheric stability; assumption: *T_mrt_* = 2·*T_a_* and *RH* = 77% (Hong Kong, humid subtropical climate (Cwa), July, at midday); R^2^: coefficient of determination

**Fig. S7** UTCI differences (ΔUTCI) between *v_10 m_*, calculated according to the logarithmic law for the VWSP from *v_h-b_* and *z_0_* = 0.01 m, and *v_10 m_*, calculated by the same method but for *z_0_* = 0.80 m, for two *v_h-b_* values; neutral atmospheric stability; assumption: *T_mrt_* = *T_a_* and *RH* = 85% (Hong Kong, humid subtropical climate (Cwa), July, at midnight); R^2^: coefficient of determination

**Table S1** Assignment of UTCI ranges to human thermal stress categories (according to Bröde et al. 2012)

| UTCI ranges (°C) | Human thermal stress categories |
| --- | --- |
| > 46.0 | extreme heat stress |
| 38.1 to 46.0 | very strong heat stress |
| 32.1 to 38.0 | strong heat stress |
| 26.1 to 32.0 | moderate heat stress |
| 9.1 to 26.0 | no thermal stress |
| 0.1 to 9.0 | slight cold stress |
| -12.9 to 0.0 | moderate cold stress |
| -26.9 to -13.0 | strong cold stress |
| -39.9 to -27.0 | very strong cold stress |
| ≤-40.0 | extreme cold stress |

**Table S2** Results for log(*z_h-b_*)⁄(*z_0_*))⁄(log(*z_10 m_*)⁄*z_0_*), *v_h-b,z0_* (for two *v_10 m_* values), and
*v*_h-b,z0_/*v*_h-b,0.01 m_, each dependent on *z_0_*

| *z_0_* (m) | log(*z_h-b_*)⁄(*z_0_*))⁄  (log(*z_10 m_*)⁄*z_0_*) | *v_h-b,z0_* (m s^-1^)  for *v_10 m_* = 1.0 m s^-1^ | *v_h-b,z0_* (m s^-1^)  for *v_10 m_* = 2.0 m s^-1^ | *v*_h-b,z0_/*v*_h-b,0.01 m_ |
| --- | --- | --- | --- | --- |
| 0.01 | 0.68 | 0.68 | 1.36 | 1.00 |
| 0.05 | 0.58 | 0.58 | 1.16 | 0.86 |
| 0.10 | 0.52 | 0.52 | 1.04 | 0.77 |
| 0.20 | 0.44 | 0.44 | 0.88 | 0.64 |
| 0.30 | 0.37 | 0.37 | 0.74 | 0.54 |
| 0.40 | 0.31 | 0.31 | 0.62 | 0.46 |
| 0.50 | 0.26 | 0.26 | 0.52 | 0.39 |
| 0.60 | 0.22 | 0.22 | 0.44 | 0.32 |
| 0.70 | 0.17 | 0.17 | 0.34 | 0.25 |
| 0.80 | 0.13 | 0.13 | 0.26 | 0.19 |

**Table S3** Results for log(*z*_10 m_)⁄(*z_0_*))⁄(log(*z_h-b_*)⁄*z_0_*), *v_10 m,z0_* (for two *v_h-b_* values), and
*v*_10 m,z0_/*v*_10 m,0.01 m_, each dependent on *z_0_*

| *z_0_* (m) | log(*z_10 m_*⁄(*z_0_*))⁄  (log(*z_h-b_*)⁄*z_0_*) | *v_10 m,z0_* (m s^-1^)  for *v_h-b_* = 0.4 m s^-1^ | *v_10 m,z0_* (m s^-1^)  for *v_h-b_* = 0.6 m s^-1^ | *v*_10 m,z0_/*v*_10 m,0.01 m_ |
| --- | --- | --- | --- | --- |
| 0.01 | 1.47 | 0.59 | 0.88 | 1.00 |
| 0.05 | 1.71 | 0.69 | 1.03 | 1.17 |
| 0.10 | 1.92 | 0.77 | 1.15 | 1.31 |
| 0.20 | 2.29 | 0.92 | 1.38 | 1.56 |
| 0.30 | 2.70 | 1.08 | 1.62 | 1.84 |
| 0.40 | 3.18 | 1.27 | 1.91 | 2.17 |
| 0.50 | 3.80 | 1.52 | 2.28 | 2.59 |
| 0.60 | 4.64 | 1.86 | 2.78 | 3.16 |
| 0.70 | 5.88 | 2.35 | 3.53 | 4.00 |
| 0.80 | 7.93 | 3.17 | 4.76 | 5.40 |

**Fig. S1** Wind speed (*v*) as a function of height (*z*) and roughness length (*z_0_*); *v_10 m_* = 1.0 m s^-1^; neutral atmospheric stability

**Fig. S2** *v_h-b,z0_*/*v_h-b,0.01 m_* and *v_10 m,z0_*/*v_10 m,0.01 m_* values dependent on the roughness length (*z_0_*); neutral atmospheric stability

**Fig. S3** Wind speed (*v*) as a function of height (*z*) and roughness length (*z_0_*); *v_h-b_* = 0.4 m s^-1^; neutral atmospheric stability

**Fig. S4** UTCI differences (ΔUTCI) between *v_10 m_*, calculated according to the logarithmic law for the VWSP from *v_h-b_* and *z_0_* = 0.01 m, and *v_10 m_*, calculated by the same method but for *z_0_* = 0.80 m, for two *v_h-b_* values; neutral atmospheric stability; assumption: *T_mrt_* = 2·*T_a_* and *RH* = 18% (Ghardaia, Algeria, hot and dry desert climate (BWh), July, at midday); R^2^: coefficient of determination

**Fig. S5** UTCI differences (ΔUTCI) between *v_10 m_*, calculated according to the logarithmic law for the VWSP from *v_h-b_* and *z_0_* = 0.01 m, and *v_10 m_*, calculated by the same method but for *z_0_* = 0.80 m, for two *v_h-b_* values; neutral atmospheric stability; assumption: *T_mrt_* = *T_a_* and *RH* = 20% (Ghardaia, Algeria, hot and dry desert climate (BWh), July, at midnight); R^2^: coefficient of determination

**Fig. S6** UTCI differences (ΔUTCI) between *v_10 m_*, calculated according to the logarithmic law for the VWSP from *v_h-b_* and *z_0_* = 0.01 m, and *v_10 m_*, calculated by the same method but for *z_0_* = 0.80 m, for two *v_h-b_* values; neutral atmospheric stability; assumption: *T_mrt_* = 2·*T_a_* and *RH* = 77% (Hong Kong, humid subtropical climate (Cwa), July, at midday); R^2^: coefficient of determination

**Fig. S7** UTCI differences (ΔUTCI) between *v_10 m_*, calculated according to the logarithmic law for the VWSP from *v_h-b_* and *z_0_* = 0.01 m, and *v_10 m_*, calculated by the same method but for *z_0_* = 0.80 m, for two *v_h-b_* values; neutral atmospheric stability; assumption: *T_mrt_* = *T_a_* and *RH* = 85% (Hong Kong, humid subtropical climate (Cwa), July, at midnight); R^2^: coefficient of determination
